# Manual therapy as a prophylactic treatment for migraine: design of a randomized controlled trial

**DOI:** 10.1186/s13063-019-3937-8

**Published:** 2019-12-27

**Authors:** Andreas Leonard Amons, Rene Franciscus Castien, Johannes C. van der Wouden, Willem De Hertogh, Joost Dekker, Henriëtte Eveline van der Horst

**Affiliations:** 1 Headache Centre at Healthcare Centre Haarlemmermeer, Waddenweg 1, 2134XL Hoofddorp, The Netherlands; 2 Department of General Practice and Elderly Care Medicine, Amsterdam Public Health Research Institute, Amsterdam University Medical Centres (location VUmc), Van der Boechorststraat 7, 1018BT Amsterdam, The Netherlands; 3 Department of Rehabilitation Sciences and Physiotherapy, Faculty of Medicine and Health Sciences, University of Antwerp, Campus Drie Eiken, Universiteitsplein 1, 2610 Wilrijk, Belgium; 4 Department of Rehabilitation Medicine, Amsterdam Public Health Research Institute, Amsterdam University Medical Centres (location VUmc), Van der Boechorststraat 7, 1018BT Amsterdam, The Netherlands; 5 Department of Psychiatry, Amsterdam Public Health Research Institute, Amsterdam University Medical Centres (location VUmc), Van der Boechorststraat 7, 1018BT Amsterdam, The Netherlands

**Keywords:** Manual therapy, Migraine, Manual pressure techniques

## Abstract

**Background:**

People with migraine often experience disability with serious consequences for their social life and work productivity. The pharmacological prophylactic management of migraine is effective in reducing migraine attacks. However, many people are reluctant to use daily prophylactic medication, leading to a demand for non-pharmacological treatment options. We present the design for and discuss the feasibility of a pragmatic, randomized controlled trial on the effectiveness of a multimodal manual therapy (MT) treatment compared to usual care by the general practitioner (GP) for the prophylactic treatment of migraine.

**Methods:**

Eligible participants will be recruited in primary care using the International Classification of Headache Disorders III criteria for migraine of the International Headache Society. Participants will be randomized to either multimodal MT treatment or usual care provided by the GP. GPs will be asked to treat the usual care group according to the Dutch GP guideline for headache. The multimodal MT intervention will include manual pressure techniques, neck muscle-strength exercises and mobilization of the cervical and thoracic spine.

The trial will consist of a 12-week treatment period and follow-up measurements at 12, 26 and 52 weeks. The primary outcome measure is the number of migraine days per 4 weeks, assessed with a headache diary. Secondary outcome measures are the number of migraine attacks, medication use, disability due to headache, headache intensity, number of participants reporting a 50% migraine reduction, measurement of cervical pressure pain thresholds, presence of allodynia, endurance of cervical flexor muscles, days of absence of work and global perceived effect.

**Discussion:**

The results of the trial will show whether a multimodal MT intervention is an effective non-pharmacological treatment option for people with migraine.

**Trial registration:**

Dutch Trial Register, NL7504. Registered on 7 February 2019.

## Background

Migraine is a common and often disabling disorder with a high impact on work, household and social life [1]. The 1-year prevalence of migraine is estimated at 15%, and migraine is ranked as the seventh-highest cause of disability in the Global Burden of Disease study [1, 2]. In Europe, the total cost of migraine is estimated at 50.000 million euro a year, making migraine the most costly headache disorder [3]. Therefore, effective treatments that reduce the frequency of migraine are highly needed [4]. The prophylactic management of migraine generally consists of pharmacological treatment [4]. Prophylactic medication (e.g., propranolol, topiramate or amitriptyline) reduces migraine attacks by 50% in 50% of patients [5].

However, taking this medication has some disadvantages. Daily intake of prophylactic medication can cause side effects, such as fatigue and dizziness, which induce some patients to refuse this medication [6]. This has led to a growing demand for non-pharmacological prophylactic treatments to reduce the frequency of migraine [7].

The results of several studies in the past decades suggest that manual therapy (MT) might be an effective treatment to reduce migraine frequency and intensity. However, some of the studies had small sample sizes and lacked appropriate randomization, allocation concealment, blinding, intention-to-treat analysis and loss to follow-up. Thus, it is not possible to draw a definite conclusion on the effectiveness of MT for migraine [8]. Also, publication bias may have favoured studies with positive results. If manual therapy is an effective treatment for the reduction of migraine attacks, it may result in a reduction of the use of drugs, which have side effects, and in a reduction of impact on personal life. Therefore, rigorous, pragmatic research is needed that is in line with the International Headache Society (IHS) guidelines for controlled trials in migraine to determine the effectiveness of MT [9].

MT treatment for the management of headaches commonly consists of mobilization and manipulation of the cervical and thoracic spine in combination with specific exercises, posture corrections and myofascial soft tissue techniques [10–12]. A multimodal MT approach, including mobilization and manipulation of the cervical spine in combination with exercise, has been reported to be effective for tension-type headache [10, 11, 13].

The pathophysiological mechanism of migraine is still not fully understood, but sensitization of the trigemino-cervical complex has been suggested to play an important role [14–18]. Bartsch and Goadsby [19] showed convergence of nociceptive afferent input by cervical dorsal roots of C1–C3 and trigeminal afferent input onto second-order neurons at the trigemino-cervical complex. This convergence of cervical and trigeminal nociception is supported by the frequent clinical presentation of people with migraine who also experience pain and allodynia in the cervical and cephalic regions [20, 21]. Manual pressure on cervical myofascial structures can provoke a typical migraine headache, indicating referred pain based on the convergence of cervical and ophthalmic nociceptive afferents at the trigemino-cervical complex [22, 23].

Decreased pressure pain thresholds have been associated with sensitization. In migraine, decreased pressure pain thresholds of the upper cervical structures and the trapezius muscle are common [24, 25]. Migraine is associated with cervical musculoskeletal dysfunction such as cervical myofascial trigger points, decreased endurance of the neck flexor muscles and restricted mobility of the upper cervical spine [23, 26, 27]. A combination of manual pressure techniques on myofascial trigger points, neck muscle strength exercises and mobilizations of the cervical and thoracic spine targets to decrease cervical nociceptive input and to reduce sensitization of the trigemino-cervical complex. We hypothesize that manual therapy can reduce the frequency of migraine by decreasing the nociceptive transmission in the trigemino-cervical complex.

The objective of our randomized controlled trial (RCT) is to assess the effectiveness of a multimodal manual therapy treatment compared to usual care for the prophylactic treatment of migraine.

## Methods

**Fig. 1:**
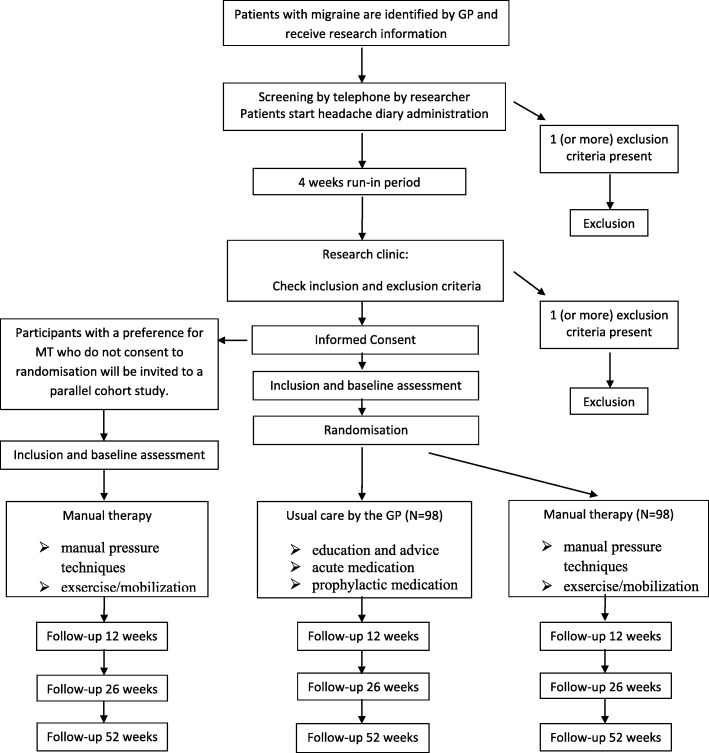
Flow diagram of the trial. GP general practitioner, MT manual therapy

This study is a single-blinded, multicentre, pragmatic clinical trial with two parallel groups assessing the potential superiority of a multimodal MT treatment over usual care by the GP. We will include a 4-week run-in period to provide accurate migraine frequency data prior to enrolment. The treatment will last 12 weeks with follow-up measurements at 12, 26 and 52 weeks (Fig. 1). The study adheres to the guidelines of the International Headache Society (IHS) for controlled trials in patients with migraine regarding inclusion criteria, outcome measurements and statistical analysis [9].

Parallel to the RCT we will conduct a prospective cohort study with migraine patients with a strong preference for MT treatment who do not want to be randomized. The aim of this parallel group is to explore the differences in patient characteristics at baseline between the randomized trial and the cohort study. Patient’s expectations with regard to recovery will be assessed in both studies and the relationship between expectations and effect of the treatment (i.e. the primary outcome measures) will be analysed. This information is necessary to better understand the generalizability of the study results. The participants in the cohort study will be treated with MT; treatment and measurements will be identical to the treatment procedure and measurements used in the RCT. All measurements will take place with a trained research assistant at one location. Participants will be given usual care by their own GP.

The design and protocol of the study have been approved by the medical ethics committee of Amsterdam University Medical Centres (location VUmc) and registered in the Dutch Trial Register (NL7504). The Standard Protocol Items: Recommendations for Interventional Trials (SPIRIT) Checklist for this research is presented in Additional file 1 [28]. Information on participants will be handled according to EU General Data Protection Regulations, and according to the guideline of the Central Committee on Research Involving Human Subjects (CCMO) in the Netherlands, to protect participant confidentiality. The study is monitored by the Clinical Research Bureau (CRB) of Amsterdam UMC location VUmc.

### Population

Participants will be recruited by the participating general practitioners (GPs) working in an urban area of Hoofddorp, The Netherlands. During consultation, the GP will provide oral and written information about the study and will invite the patient to participate. If the patient is interested in participating in the study and consents to providing contact details to the researcher, the GP notifies the researcher by email. The researcher will provide additional information about the study to the participant, followed by a telephone interview after 1 week to answer possible questions and check the inclusion and exclusion criteria. Information on the study will be provided by posters and folders in participating general practices and at the website of the coordinating healthcare centre.

### Inclusion criteria

Eligible participants are between 18 and 65 years of age and should have had migraine attacks for more than 1 year, according to the diagnostic criteria of the International Classification of Headache Disorders (ICHD) III [29]. A GP or neurologist should have established the diagnosis of migraine, and the frequency of attacks should be two times a month or more. Co-occurrence of tension-type headache is allowed if the participant can clearly distinguish this headache from migraine. Participants will only be included if they have concomitant neck pain between migraine attacks or during an attack. The use of prophylactic medication is allowed if migraine is stable and medication use has not changed in the last 3 months. Furthermore, participants have to be able to read and write Dutch.

Exclusion criteria are (suspected) malignancy, pregnancy, cerebrovascular disease, degenerative central nervous system diseases, medication-overuse headache, current diagnosis of depression or other severe psychiatric disease, rheumatoid arthritis, serious or systemic infection, fever or change in medication for migraine within 3 months before the study, and having received MT treatment for migraine up to 3 months prior to the start of the study.

### Data collection

Participants are asked to keep a headache diary and will be instructed on the way to report, in order to obtain baseline data for migraine characteristics, and will receive an appointment with the research assistant after 4 weeks. Headache diaries will be provided on paper. On each day, participants are able to report: no headache, tension-type headache or migraine, medication use and absence of work because of migraine. Before baseline measurement, the research assistant will check the inclusion and exclusion criteria and ask for written informed consent. Four weeks before the follow-up appointment, participants will be asked by email or telephone to start filling out a headache diary.

At all measurements, the data will be collected electronically (Castor EDC). The secured electronic program includes data validation checks and a data audit trail according to Good Clinical Practice standards. Only the researchers and the research assistant will have access to the data. Information on participants will be handled according to privacy regulations of the Amsterdam Public Health Quality Handbook (http://www.emgo.nl/kc/privacy/).

### Baseline assessment

Baseline assessment will include the registration of demographic variables (age, gender, education and profession), migraine characteristics according to ICHD III criteria, other physical complaints and chronic diseases.

**Fig. 2:**
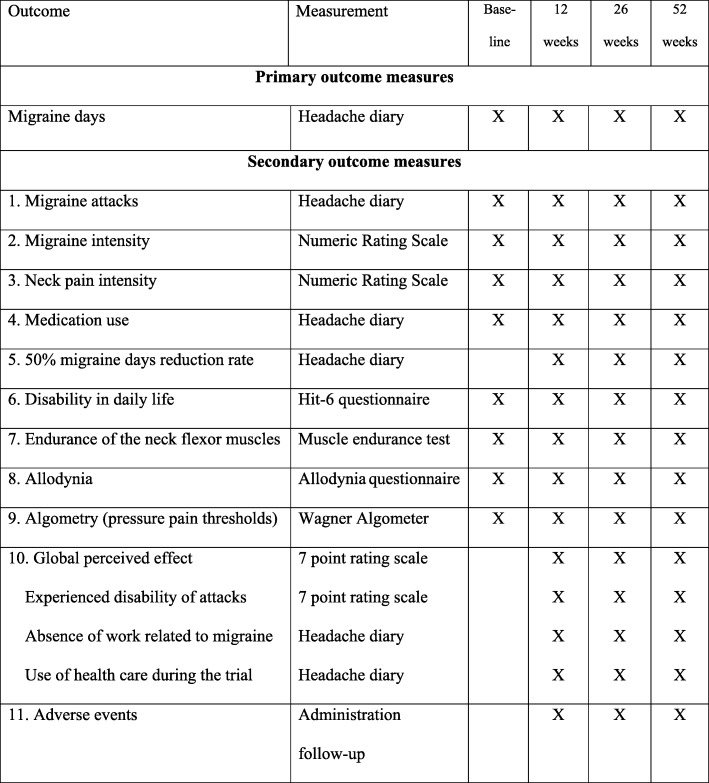
Schedule of outcome measures and assessments. HIT-6, Headache Impact Test

Expectations regarding the effectiveness of treatment will be measured on a 7-point rating scale (range from 0 = no result to 6 = excellent result expected). Patient’s preference for treatment will be administrated (preference for usual care, MT or no preference). Other outcome measures include disability (Headache Impact Test questionnaire (HIT-6)) [30], allodynia (allodynia questionnaire) [31], pressure pain thresholds [32] and neck flexor muscle endurance [33] (see later for details). Figure 2 shows all outcome measures and assessments.

### Randomization

After baseline measurement, randomization will take place with a 1:1 allocation ratio. An independent statistician who has no involvement with the clinical investigators will generate a random sequence of numbers before the start of the study. The research assistant who is blinded for the randomization sequence will supply sealed and numbered envelopes. In the presence of another administrative assistant, the participant will open the sealed envelope, and an appointment will be made for treatment by either the participating GP or a manual therapist. Participants will be invited to a parallel cohort study if a strong preference for MT treatment keeps them from agreeing to randomization.

### Blinding

Allocation of participants is concealed from the researcher and the research assistant who performs all of the measurements. An independent statistician will carry out the statistical analysis and review the interpretation of the results. For obvious reasons, participating patients, GPs and manual therapists cannot be blinded to treatment.

### Usual care

Participants assigned to the usual care group will be treated by their general practitioner (GP). The GP will treat participants as usual, based on the recommendations of the practice guideline for headache of the Dutch College of General Practitioners [34]. The GP provides lifestyle advice and, if necessary, prescribes medication. The recommended treatment consists of acute medication for a single attack or prophylactic medication when the migraine attacks occur two times a month or more [5]. The GP will evaluate the treatment in consecutive appointments. Participating GPs will be informed about the research protocol by the researcher during a 1-h meeting.

### Intervention

The multimodal manual therapy (MT) treatment aims at restoring cervical function in order to reduce nociceptive cervical afferent output. The treatment will include manual pressure techniques on the trapezius muscle and upper cervical/suboccipital musculature to decrease neck pain intensity and cervical muscle tenderness [35]. Neck muscle strength will be trained, by giving low-load craniocervical muscle exercises and correcting sitting and standing postures [36]. The selected spinal mobilizations are low and high-velocity techniques of the cervical and thoracic spine. To create a protocol that will be feasible for all participants, no high-velocity thrust manipulations of the upper cervical region (C0–C3) will be applied in the study, due to possible individual risk factors associated with serious adverse events [37, 38].

Experienced manual therapists will be trained in the treatment protocol prior to the study. The treatment protocol provides recommendations of techniques that can be used; the treating manual therapist decides which techniques will be included, depending on the condition of the participant. The applied techniques will be documented for each session on an evaluation form. Instructions on posture and home exercises will be provided to the participants in booklets. The MT intervention will consist of a maximum of nine sessions of 30 min each, starting with treatment once a week, followed by once every other week during 12 weeks.

During the 12 weeks of treatment, participants will be asked not to make use of additional therapies or medication for their migraine. At all follow-up measurements, possible use of additional therapies and medication will be asked for and registered.

### Primary outcome measure

The primary outcome of the study is the number of migraine days, recorded by the participant in a headache diary during all follow-up assessments [9]. A migraine day is defined as a day with migraine characteristics according to the IHS classification ICDH III for longer than 4 h, or a headache that resolves with the intake of triptans or ergotamine within 2 h of intake [29].

### Secondary outcome measures

The secondary outcome measures are as follows:

- Number of migraine attacks per 4 weeks, recorded in a headache diary during the 4 weeks before follow-up measurements [9]. Migraine attacks will be considered separate attacks if 48 h without headache is reported in the headache diary.
- Intensity of migraine, assessed on an 11-point numerical rating scale (0 = no pain, 10 = most severe pain) [39].
- Intensity of neck pain, assessed on an 11-point numerical rating scale (0 = no pain, 10 = most severe pain) [39].
- Medication use as number of doses per 4 weeks of simple analgesics (e.g., paracetamol), NSAIDs, acute migraine medication (triptans and ergotamines) or prophylactic medication. Participants are asked to report changes of medication to the research assistant at all follow-up measurements.
- Responder rate will be measured by the number of migraine days before vs. after treatment, dichotomized into ≥ 50% reduction or not [9].
- Disability, assessed by the HIT-6 questionnaire. The HIT-6 questionnaire consists of six questions measuring pain intensity, social functioning, role functioning, vitality, cognitive functioning and psychological distress on a 5-point ordinal rating scale (never to always). Internal consistency is considered high (Cronbach’s α = 0.82–0.90) and test–retest reliability is fair (ICC = 0.77) [30]. The Dutch version of the HIT-6 questionnaire has shown to be a valid and reliable tool to measure the impact of migraine [40].
- The endurance of the neck flexor muscles will be scored as the number of seconds the participant can raise his head from the table when lying in a supine position, as described by Harris et al. [33]. Harris et al. reported good to excellent intra-tester reliability (ICC = 0.82–0.91) and moderate inter-tester reliability (ICC = 0.67–0.78) [33].
- Cutaneous allodynia (CA) will be evaluated with the 12-item allodynia symptom checklist. This questionnaire consists of 12 questions about cutaneous hypersensitivity in the cervical cephalic region. The participant can score yes, no or not applicable. Allodynia symptoms and score on CA severity are defined in the following categories: none (0–2), mild (3–5), moderate (6–8) and severe (9 or higher) [31].
- We will perform algometry, by measuring pressure pain thresholds (PPTs) with a Wagner FDK algometer at the upper trapezius muscle (at the midpoint between C7 spinosus and the acromion), the suboccipital muscles and the anterior tibial muscle. The PPT measurement will be repeated three times at each point, and a mean score will be calculated. Algometry has demonstrated excellent intra-tester reliability (upper trapezius test–retest ICC = 0.83, 95% CI 0.69–0.91), and excellent inter-tester reliability (upper trapezius ICC = 0.89, 95% CI 0.83–0.93) [32].
- Participants will be asked to report the global perceived effect on a 7-point rating scale (0 = much worse to 6 = much better). Disability due to attacks will be assessed on a 5-point rating scale (0 = no disability and no medication to 4 = fully disabled even with medication). Also, use of healthcare resources and absence of work will be reported.
- All adverse events will be administrated for both treatments at all follow-up measurements.

### Statistical analysis

Baseline characteristics will be presented as percentages for categorical variables, and as means and standard deviations for continuous data, using descriptive statistics. The distribution of the data will be evaluated using histograms and Q–Q plots. The outcomes will be adjusted for baseline differences. The outcomes of the total follow-up period, including baseline data, will be examined with a linear mixed-model analysis. Differences between groups will be reported and shown in tables. Differences between the cohort group and the RCT group (separate and combined) will be analysed with Student *t* tests (continuous data) and chi-squared tests (nominal data). For non-parametric data, the Mann–Whitney *U* test will be used. The primary analysis will be by intention to treat. Additionally, a per-protocol analysis will be carried out to assess the effect in participants who adhered to the protocol. Protocol adherence will be defined as staying in the allocated treatment group during the 12-week treatment period; for MT treatment, participants have to complete at least six sessions. In the ‘usual care’ group, participants who receive MT during the trial period will be excluded from the per-protocol analysis. Participants will also be excluded from the per-protocol analysis if they report the use of additional healthcare for migraine during the trial period. Effect sizes will be computed for normally distributed outcomes. Statistical analysis will be carried out using SPSS version 23 (IBM Corporation, Armonk, NY, USA).

### Sample size

Taking pilot study results as the basis for our calculation, we assume an average frequency of 4.2 migraine days (SD = 2.4). As we want to detect a difference in the reduction of the number of migraine days of at least 25% between groups, with a two-sided significance level of 0.05 and a power of 0.80, each group will have to include 83 evaluable persons. Taking into account a 15% loss to follow-up, a total of (100 / 85) × 83 × 2 = 196 participants will have to be enrolled into the study, 98 per group.

To ensure the enrolment of the required number of participants over an estimated period of 2 years, we will recruit 44 GPs and four manual therapists to participate in the full trial.

### Feasibility of the study

We performed a pilot study to assess the feasibility of the measurements, the treatment protocol and randomization procedures. The pilot study concerned 24 possible participants in 8 weeks (October–December 2015); 11 participants fulfilled the inclusion criteria.

Two out of 13 excluded participants had a strong preference for manual therapy treatment and, therefore, were excluded from randomization. Other reasons for exclusion were: no migraine according to the IHS criteria, low frequency of migraine and participants with GPs who did not participate in the pilot study. The research protocol was evaluated by questionnaires and in personal meetings with the participating manual therapists, GPs, research assistant and participants. GPs and manual therapists reported no problems with adhering to the protocol for measurements and treatment. The results of the pilot study showed that the treatment protocol and procedures were feasible and that the participants tolerated both treatments well.

## Discussion

We have described the design of an RCT to assess the effectiveness of a multimodal MT intervention for the treatment of migraine. We performed a pilot study to evaluate the feasibility of our protocol and procedures. The results of this pilot were encouraging; the expected recruitment was accomplished within a period of 8 weeks, and participants tolerated the MT treatment protocol and measurements without problems.

MT is a commonly used non-pharmacological treatment for migraine in primary care [12]; however, the evidence of MT to reduce migraine attacks is scarce and shows methodological flaws. Therefore, with this trial, we attempt to strengthen the evidence and to minimize the methodological shortcomings.

A strength of our design is that we adhere to the clinical trial guideline of the IHS concerning the inclusion and exclusion criteria for migraine and statistical analysis. This will make it possible to provide a more robust conclusion on the effectiveness of the MT.

Although the IHS guidelines recommend both the number of migraine days and the number of migraine attacks as the primary outcome, we have included only one primary outcome: the number of migraine days. To report one primary outcome is in line with the CONSORT Statement [41]. Furthermore, we believe that the number of migraine days is a relevant clinical outcome and will be more responsive to change.

A strength of this study is that we compare state-of-the-art, guideline-based usual care with a new treatment option. Moreover, a pragmatic trial with two regularly applied treatments will enhance external validity of the results [42] and is in line with other studies [36, 43].

Our study has a few limitations. One of the limitations concerns the absence of blinding of participants, manual therapists and GPs. Furthermore, the participant will receive information about the treatment and the intended goal, which may lead to information bias.

We did not include a placebo or sham manual therapy treatment as a control intervention but chose to compare two active and commonly used treatment interventions in a primary care setting. We argue that it would be unethical to withhold an effective prophylactic treatment as a comparator for patients with frequent migraine. Additionally, we will not be able to control for differences in given attention during treatment and placebo effects that are reported in manual therapy research [44].

In this study, we will use the 12-item allodynia symptom checklist to evaluate cutaneous allodynia. This checklist is validated using quantitative sensory testing as a gold standard but needs further validation for reliability and responsiveness [31].

The pilot study showed that 15% of the participants had a preference for the MT treatment, which withheld participants from being randomized. Separate from the RCT, a parallel cohort study will be conducted for this group to compare these results with the RCT outcomes. Expectations regarding treatment outcome will be assessed in both the RCT and the cohort study because these could influence differences in outcomes.

The results of this study will be published in peer-reviewed journals in agreement with the CONSORT 2010 Statement [41]. Furthermore, results will be provided to the Dutch Association of Headache Patients, the Dutch journal for general practitioners and the journal for physiotherapists in the Netherlands. This study aims to produce evidence pertaining to non-pharmacological prophylactic treatments for migraine. The results of this study may support patients and GPs in their decision-making in the search for prophylactic treatment options to reduce the burden that migraine has on personal life and society.

## Trial status

Protocol version 5, 13 June 2019. The study is in the recruitment phase. The recruitment period is estimated from 8 April 2019 to May 2021.

## Supplementary information

**Additional file 1.** SPIRIT 2013 Checklist.

## Data Availability

The research data are owned by VUmc. The researchers of the research group at VUmc will have access to the final data as long as there is a contractual agreement of the researcher with Amsterdam UMC, location VUmc. Access to the protocol, the dataset and statistical codes will be granted after closure of the study.

## Abbreviations

Amsterdam UMC: Amsterdam University Medical Centres
CA: Cutaneous allodynia
CRB: Clinical Research Bureau
GP: General practitioner
ICHD: International Classification of Headache Disorders
IHS: International Headache Society
MT: Manual therapy
PPT: Pressure pain threshold
RCT: Randomized controlled trial
VUmc: Vrije Universiteit, Medical Centre
