# Supplementary material for: Manual therapy as a prophylactic treatment for migraine: design of a randomized controlled trial
Source: Trials. 2019 Dec 27;20:785. doi: 10.1186/s13063-019-3937-8 (PMC6935086; doi:10.1186/s13063-019-3937-8)
Supplement: Supplementary file 1 — Additional file 1. SPIRIT 2013 Checklist. [file 13063_2019_3937_MOESM1_ESM.doc]

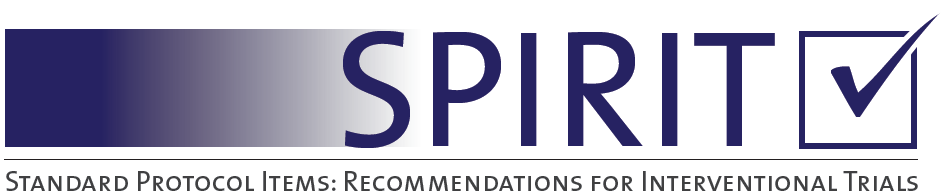


SPIRIT 2013 Checklist: Recommended items to address in a clinical trial protocol and related documents*

| Section/item | ItemNo | Description | Section available |  |  |
| --- | --- | --- | --- | --- | --- |
| **Administrative information** | | |  |  |  |
| Title | 1 | Descriptive title identifying the study design, population, interventions, and, if applicable, trial acronym | Title  page 1 |  |  |
| Trial registration | 2a | Trial identifier and registry name. If not yet registered, name of intended registry | Abstract  page 2 |  |  |
|  | 2b | All items from the World Health Organization Trial Registration Data Set | Provided in methods section  pp. 5-14 and at www.trialregister.nl/trial/7504 |  |  |
| Protocol version | 3 | Date and version identifier | Registration details provided in trial register (page 2) and at  www.trialregister.nl/trial/7504 |  |  |
| Funding | 4 | Sources and types of financial, material, and other support | Provided at the end of main manuscript in the declarations section  page 17 |  |  |
| Roles and responsibilities | 5a | Names, affiliations, and roles of protocol contributors | Provided at the end of main manuscript in the declarations section  page 17 |  |  |
|  | 5b | Name and contact information for the trial sponsor | Not applicable |  |  |
|  | 5c | Role of study sponsor and funders, if any, in study design; collection, management, analysis, and interpretation of data; writing of the report; and the decision to submit the report for publication, including whether they will have ultimate authority over any of these activities | Provided at the end of main manuscript in the declarations section  page 17 |  |  |
|  | 5d | Composition, roles, and responsibilities of the coordinating centre, steering committee, endpoint adjudication committee, data management team, and other individuals or groups overseeing the trial, if applicable (see Item 21a for data monitoring committee) | The research protocol is approved by the METc (ethics committee) and by the APH research institute. The research group at Amsterdam UMC, location Vrije Universiteit medical centre  (VUmc) is responsible for the protocol decisions. As the executive researcher, A. Amons is responsible for form development, database development, data collection and data management, supervised by the other members of the research group: dr. R. Castien (local supervision); dr. H van der Wouden (supervisor at Amsterdam UMC); prof. dr. J. Dekker and prof. dr. H. van der Horst. The data analysis will be performed by an independent statistician of Amsterdam UMC, location VUmc.  Quality control is governed by the Clinical Research Bureau (CRB) and the ethics committee of VUmc. There are no additional committees. |  |  |
| Introduction |  |  |  |  |  |
| Background and rationale | 6a | Description of research question and justification for undertaking the trial, including summary of relevant studies (published and unpublished) examining benefits and harms for each intervention | Provided in the background section  page 3-5 |  |  |
|  | 6b | Explanation for choice of comparators | Provided in the background section  page 3 |  |  |
| Objectives | 7 | Specific objectives or hypotheses | Provided in the background section  page 4-5 |  |  |
| Trial design | 8 | Description of trial design including type of trial (eg, parallel group, crossover, factorial, single group), allocation ratio, and framework (eg, superiority, equivalence, noninferiority, exploratory) | Provided in the methods section  page 5 |  |  |
| Methods: Participants, interventions, and outcomes | | |  |  |  |
| Study setting | 9 | Description of study settings (eg, community clinic, academic hospital) and list of countries where data will be collected. Reference to where list of study sites can be obtained | Provided in the methods section  page 6 |  |  |
| Eligibility criteria | 10 | Inclusion and exclusion criteria for participants. If applicable, eligibility criteria for study centres and individuals who will perform the interventions (eg, surgeons, psychotherapists) | Provided in the methods section  page 6-7 |  |  |
| Interventions | 11a | Interventions for each group with sufficient detail to allow replication, including how and when they will be administered | Provided in the methods section  page 9-10 |  |  |
|  | 11b | Criteria for discontinuing or modifying allocated interventions for a given trial participant (eg, drug dose change in response to harms, participant request, or improving/worsening disease) | Provided in methods section  page 9-10 |  |  |
|  | 11c | Strategies to improve adherence to intervention protocols, and any procedures for monitoring adherence (eg, drug tablet return, laboratory tests) | Provided in the methods section  page 10 |  |  |
|  | 11d | Relevant concomitant care and interventions that are permitted or prohibited during the trial | Provided in the methods section  page 6 and 10 |  |  |
| Outcomes | 12 | Primary, secondary, and other outcomes, including the specific measurement variable (eg, systolic blood pressure), analysis metric (eg, change from baseline, final value, time to event), method of aggregation (eg, median, proportion), and time point for each outcome. Explanation of the clinical relevance of chosen efficacy and harm outcomes is strongly recommended | Provided in the methods section  page 10-12 and figure 2 |  |  |
| Participant timeline | 13 | Time schedule of enrolment, interventions (including any run-ins and washouts), assessments, and visits for participants. A schematic diagram is highly recommended (see Figure) | Provided in the methods section (page 5) and Figure 1 and Figure 2 |  |  |
| Sample size | 14 | Estimated number of participants needed to achieve study objectives and how it was determined, including clinical and statistical assumptions supporting any sample size calculations | Provided in the methods section at section Sample size  page 13 |  |  |
| Recruitment | 15 | Strategies for achieving adequate participant enrolment to reach target sample size | Potential participants are identified and recruited by the general practitioner (GP). Participating GPs are informed about the procedures in a meeting and every two months by a newsletter. Information of the study for participants will be provided by posters, booklets and websites of participating general practices. Participants apply for participation during consultation by the GP. All participants need to have a referral from the GP; no other methods are used |  |  |
| **Methods: Assignment of interventions (for controlled trials)** | | |  |  |  |
| Allocation: |  |  |  |  |  |
| Sequence generation | 16a | Method of generating the allocation sequence (eg, computer-generated random numbers), and list of any factors for stratification. To reduce predictability of a random sequence, details of any planned restriction (eg, blocking) should be provided in a separate document that is unavailable to those who enrol participants or assign interventions | Provided in the methods section  page 8 |  |  |
| Allocation concealment mechanism | 16b | Mechanism of implementing the allocation sequence (eg, central telephone; sequentially numbered, opaque, sealed envelopes), describing any steps to conceal the sequence until interventions are assigned | Provided in the methods section  page 8 |  |  |
| Implementation | 16c | Who will generate the allocation sequence, who will enrol participants, and who will assign participants to interventions | Provided in the methods section  page 8 |  |  |
| Blinding (masking) | 17a | Who will be blinded after assignment to interventions (eg, trial participants, care providers, outcome assessors, data analysts), and how | Provided in the methods section  page 9 |  |  |
|  | 17b | If blinded, circumstances under which unblinding is permissible, and procedure for revealing a participant’s allocated intervention during the trial | Not applicable |  |  |
| **Methods: Data collection, management, and analysis** | | |  |  |  |
| Data collection methods | 18a | Plans for assessment and collection of outcome, baseline, and other trial data, including any related processes to promote data quality (eg, duplicate measurements, training of assessors) and a description of study instruments (eg, questionnaires, laboratory tests) along with their reliability and validity, if known. Reference to where data collection forms can be found, if not in the protocol | Provided in the methods section  page 12-13 |  |  |
|  | 18b | Plans to promote participant retention and complete follow-up, including list of any outcome data to be collected for participants who discontinue or deviate from intervention protocols | Provided in the methods section  page 7 |  |  |
| Data management | 19 | Plans for data entry, coding, security, and storage, including any related processes to promote data quality (eg, double data entry; range checks for data values). Reference to where details of data management procedures can be found, if not in the protocol | At all measurements, data will be collected electronically using Castor EDC. The VUmc has a campus licence for Castor EDC. Castor EDC is a secured cloud-based programme which adheres to EU General Data Protection Regulations and to Good Clinical Practice (GCP) standards. Quality control is performed by the Clinical Research Bureau as is stated in the reply on comment 1. Only the researchers and the research assistant will have access to the data.  Data will be entered and checked for missing values by the research assistant at the healthcare center. In addition, Castor EDC will perform data validation checks and provides a data audit trail. Additionally, the executing researcher performs a data check every month and reports his findings to the research assistant for clarification. The research assistant will perform error correction. |  |  |
| Statistical methods | 20a | Statistical methods for analysing primary and secondary outcomes. Reference to where other details of the statistical analysis plan can be found, if not in the protocol | Provided in the methods section  page 12-13 |  |  |
|  | 20b | Methods for any additional analyses (eg, subgroup and adjusted analyses) | Provided in the methods section  page 12-13 |  |  |
|  | 20c | Definition of analysis population relating to protocol non-adherence (eg, as randomised analysis), and any statistical methods to handle missing data (eg, multiple imputation) | Provided in the methods section  page 13 |  |  |
| **Methods: Monitoring** | | |  |  |  |
| Data monitoring | 21a | Composition of data monitoring committee (DMC); summary of its role and reporting structure; statement of whether it is independent from the sponsor and competing interests; and reference to where further details about its charter can be found, if not in the protocol. Alternatively, an explanation of why a DMC is not needed | Available on request to authors |  |  |
|  | 21b | Description of any interim analyses and stopping guidelines, including who will have access to these interim results and make the final decision to terminate the trial | Not applicable |  |  |
| Harms | 22 | Plans for collecting, assessing, reporting, and managing solicited and spontaneously reported adverse events and other unintended effects of trial interventions or trial conduct | Provided in methods section  page 12 and in figure 2 |  |  |
| Auditing | 23 | Frequency and procedures for auditing trial conduct, if any, and whether the process will be independent from investigators and the sponsor | Provided at the method section page 6 |  |  |
| Ethics and dissemination | | |  |  |  |
| Research ethics approval | 24 | Plans for seeking research ethics committee/institutional review board (REC/IRB) approval | Ethical approval obtained and available on request to authors |  |  |
| Protocol amendments | 25 | Plans for communicating important protocol modifications (eg, changes to eligibility criteria, outcomes, analyses) to relevant parties (eg, investigators, REC/IRBs, trial participants, trial registries, journals, regulators) | The research group will discuss if important protocol modifications are necessary. Subsequently, the proposed changes will be submitted and reviewed by the Medical Ethical Review Board of Amsterdam UMC, location VUmc and, on approval, adjusted in the Dutch Trial Register.  The research assistant, all general practitioners and manual therapists will be informed by email and newsletters about the modifications.  If changes are relevant for participants who are already engaged in the study, participants will be informed by email. |  |  |
| Consent or assent | 26a | Who will obtain informed consent or assent from potential trial participants or authorised surrogates, and how (see Item 32) | Provided in methods section  page 7 |  |  |
|  | 26b | Additional consent provisions for collection and use of participant data and biological specimens in ancillary studies, if applicable | Not applicable |  |  |
| Confidentiality | 27 | How personal information about potential and enrolled participants will be collected, shared, and maintained in order to protect confidentiality before, during, and after the trial | Provided in the manuscript at Method section page 6 |  |  |
| Declaration of interests | 28 | Financial and other competing interests for principal investigators for the overall trial and each study site | Provided in the manuscript at section Declarations  page 18 |  |  |
| Access to data | 29 | Statement of who will have access to the final trial dataset, and disclosure of contractual agreements that limit such access for investigators | Provided in the manuscript at section declarations  page 18 |  |  |
| Ancillary and post-trial care | 30 | Provisions, if any, for ancillary and post-trial care, and for compensation to those who suffer harm from trial participation | Provided in the manuscript at section declarations  page 18 |  |  |
| Dissemination policy | 31a | Plans for investigators and sponsor to communicate trial results to participants, healthcare professionals, the public, and other relevant groups (eg, via publication, reporting in results databases, or other data sharing arrangements), including any publication restrictions | Provided in the discussion paragraph page 17. |  |  |
|  | 31b | Authorship eligibility guidelines and any intended use of professional writers | Provided in the manuscript at section declarations  page 18 |  |  |
|  | 31c | Plans, if any, for granting public access to the full protocol, participant-level dataset, and statistical code | Provided in the manuscript at section declarations  page 18 |  |  |
| Appendices |  |  |  |  |  |
| Informed consent materials | 32 | Model consent form and other related documentation given to participants and authorised surrogates | Available on request to authors |  |  |
| Biological specimens | 33 | Plans for collection, laboratory evaluation, and storage of biological specimens for genetic or molecular analysis in the current trial and for future use in ancillary studies, if applicable | Not applicable |  |  |
